# Supplementary material for: Crawling and Gliding: A Computational Model for Shape-Driven Cell Migration
Source: PLoS Comput Biol. 2015 Oct 21;11(10):e1004280. doi: 10.1371/journal.pcbi.1004280 (PMC4619082; doi:10.1371/journal.pcbi.1004280)
Supplement: S1 Code — (ZIP) [file pcbi.1004280.s012.zip › release/tst/doc/html/classQtGraphics.html]

Tissue Simulation Toolkit: QtGraphics Class Reference


Tissue Simulation Toolkit
 0.1.4.1


- Main Page
- Namespaces
- Classes
- Files

- Class List
- Class Hierarchy
- Class Members

Public Slots |
Signals |
Public Member Functions |
List of all members

QtGraphics Class Reference

`#include <qtgraph.h>`

Inheritance diagram for QtGraphics:

[legend]

Collaboration diagram for QtGraphics:

[legend]

|  |  |
| --- | --- |
| Public Slots | |
| void | TimeStepWrap (void) |
|  | |

|  |  |
| --- | --- |
| Signals | |
| void | SimulationDone (void) |
|  | |

|  |  |
| --- | --- |
| Public Member Functions | |
|  | QtGraphics (int xfield, int yfield, const char \*movie\_file=0) |
|  | |
|  | QtGraphics (QWidget \*parent, const char \*name, int xfield, int yfield, const char \*movie\_file=0) |
|  | |
| virtual | ~QtGraphics (void) |
|  | |
| virtual void | BeginScene (void) |
|  | BeginScene() must be called before calling drawing functions. More... |
|  | |
| virtual void | EndScene (void) |
|  | EndScene() must be called to flush the drawing buffer and display the scene. More... |
|  | |
| virtual void | Point (int colour, int i, int j) |
|  | Plot a point in the Graphics window. More... |
|  | |
| virtual void | Line (int x1, int y1, int x2, int y2, int colour) |
|  | Draws a line (obviously... :-) More... |
|  | |
| virtual int | GetXYCoo (int \*X, int \*Y) |
|  | Probes the Window for user interaction, with mouse or keyboard. More... |
|  | |
| virtual int | XField (void) const |
|  | Returns the width of the Graphics window, in pixels. More... |
|  | |
| virtual int | YField (void) const |
|  | Returns the height of the Graphics window, in pixels. More... |
|  | |
| virtual void | Write (char \*fname, int quality=-1) |
|  | Writes the Image to a file. More... |
|  | |
| void | ClearImage (void) |
|  | |
| virtual void | TimeStep (void) |
|  | Implement this member function in your simulation code. More... |
|  | |
| virtual void | resizeEvent (QResizeEvent \*event) |
|  | |
| Public Member Functions inherited from Graphics | |
| virtual | ~Graphics (void) |
|  | |
| virtual void | Field (const int \*\*f, int mag=1) |
|  | Plots a field of values given by \*\*f, using color coding given by colormap file. More... |
|  | |

## Constructor & Destructor Documentation

|  |  |  |  |
| --- | --- | --- | --- |
| QtGraphics::QtGraphics | ( | int | *xfield*, |
|  |  | int | *yfield*, |
|  |  | const char \* | *movie\_file* = `0` |
|  | ) |  |  |

Referenced by QtGraphics().

|  |  |  |  |
| --- | --- | --- | --- |
| QtGraphics::QtGraphics | ( | QWidget \* | *parent*, |
|  |  | const char \* | *name*, |
|  |  | int | *xfield*, |
|  |  | int | *yfield*, |
|  |  | const char \* | *movie\_file* = `0` |
|  | ) |  |  |

inline

References QtGraphics().

|  |  |  |  |  |  |
| --- | --- | --- | --- | --- | --- |
| QtGraphics::~QtGraphics | ( | void |  | ) |  |

virtual

## Member Function Documentation

|  |  |  |  |  |  |
| --- | --- | --- | --- | --- | --- |
| void QtGraphics::BeginScene | ( | void |  | ) |  |

virtual

BeginScene() must be called before calling drawing functions.

Reimplemented from Graphics.

|  |  |  |  |  |  |
| --- | --- | --- | --- | --- | --- |
| void QtGraphics::ClearImage | ( | void |  | ) |  |

inline

|  |  |  |  |  |  |
| --- | --- | --- | --- | --- | --- |
| void QtGraphics::EndScene | ( | void |  | ) |  |

virtual

EndScene() must be called to flush the drawing buffer and display the scene.

Reimplemented from Graphics.

|  |  |  |  |
| --- | --- | --- | --- |
| int QtGraphics::GetXYCoo | ( | int \* | *X*, |
|  |  | int \* | *Y* |
|  | ) |  |  |

virtual

Probes the Window for user interaction, with mouse or keyboard.

This function should return immediately, and return 0 if there was no user interaction.

Parameters
:   |  |  |
    | --- | --- |
    | \*X,\*Y | Pointer where the clicked coordinate will be stored. |

Implements Graphics.

|  |  |  |  |
| --- | --- | --- | --- |
| void QtGraphics::Line | ( | int | *x1*, |
|  |  | int | *y1*, |
|  |  | int | *x2*, |
|  |  | int | *y2*, |
|  |  | int | *colour* |
|  | ) |  |  |

virtual

Draws a line (obviously... :-)

Parameters
:   |  |  |
    | --- | --- |
    | x1,y1 | First coordinate pair. |
    | x2,y2 | Second coordinate pair. |
    | color | Color of the line, as given in the colormap file "default.ctb". |

Implements Graphics.

|  |  |  |  |
| --- | --- | --- | --- |
| void QtGraphics::Point | ( | int | *color*, |
|  |  | int | *x*, |
|  |  | int | *y* |
|  | ) |  |  |

virtual

Plot a point in the Graphics window.

Parameters
:   |  |  |
    | --- | --- |
    | color | Color index, as defined in colormap file "default.ctb", which should be in the same directory as the executable. |
    | x,y | Coordinate of point, in Graphics coordinates (typically twice as large as the cellular automata coordinates). |

Implements Graphics.

|  |  |  |  |  |  |
| --- | --- | --- | --- | --- | --- |
| void QtGraphics::resizeEvent | ( | QResizeEvent \* | *event* | ) |  |

virtual

|  |  |  |  |  |  |
| --- | --- | --- | --- | --- | --- |
| void QtGraphics::SimulationDone | ( | void |  | ) |  |

signal

|  |  |  |  |  |  |
| --- | --- | --- | --- | --- | --- |
| virtual void QtGraphics::TimeStep | ( | void |  | ) |  |

virtual

Implement this member function in your simulation code.

Include all actions that should be carried out during a simulation step, including PDE and CPM simulation steps. See the included examples (vessel.cpp, sorting.cpp) for more information.

Reimplemented from Graphics.

|  |  |  |  |  |  |
| --- | --- | --- | --- | --- | --- |
| void QtGraphics::TimeStepWrap | ( | void |  | ) |  |

slot

References Parameter::mcs, and par.

|  |  |  |  |
| --- | --- | --- | --- |
| void QtGraphics::Write | ( | char \* | *fname*, |
|  |  | int | *quality* = `-1` |
|  | ) |  |  |

virtual

Writes the Image to a file.

File format is inferred from file extension. Currently only PNG is supported by the X-Windows implementation; the Qt-implentation supports all formats supported by Qt.

Parameters
:   |  |  |
    | --- | --- |
    | fname | File name with standard image file extension (e.g. png). |
    | quality | Quality of JPEG images, defaults to -1 (no value provided). |

Implements Graphics.

|  |  |  |  |  |  |
| --- | --- | --- | --- | --- | --- |
| virtual int QtGraphics::XField | ( | void |  | ) | const |

inlinevirtual

Returns the width of the Graphics window, in pixels.

Reimplemented from Graphics.

|  |  |  |  |  |  |
| --- | --- | --- | --- | --- | --- |
| virtual int QtGraphics::YField | ( | void |  | ) | const |

inlinevirtual

Returns the height of the Graphics window, in pixels.

Reimplemented from Graphics.

---

The documentation for this class was generated from the following files:

- qtgraph.h
- qt3graph.cpp
- qtgraph.cpp


---

Generated on Thu Aug 14 2014 22:04:01 for Tissue Simulation Toolkit by  

 1.8.6
